# The phosphotransferase VanU represses expression of four *qrr* genes antagonizing VanO-mediated quorum-sensing regulation in *Vibrio anguillarum*

**DOI:** 10.1099/mic.0.051011-0

**Published:** 2011-12

**Authors:** Barbara Weber, Kristoffer Lindell, Samir El Qaidi, Erik Hjerde, Nils-Peder Willassen, Debra L. Milton

**Affiliations:** 1 Department of Molecular Biology, Umeå Centre for Microbial Research (UCMR), Umeå University, Umeå SE-901 87, Sweden; 2 Department of Chemistry, Faculty of Science and Technology, University of Tromsø, Tromsø 9037, Norway

## Abstract

*Vibrio anguillarum* utilizes quorum sensing to regulate stress responses required for survival in the aquatic environment. Like other *Vibrio* species, *V. anguillarum* contains the gene *qrr1*, which encodes the ancestral quorum regulatory RNA Qrr1, and phosphorelay quorum-sensing systems that modulate the expression of small regulatory RNAs (sRNAs) that destabilize mRNA encoding the transcriptional regulator VanT. In this study, three additional Qrr sRNAs were identified. All four sRNAs were positively regulated by σ^54^ and the σ^54^-dependent response regulator VanO, and showed a redundant activity. The Qrr sRNAs, together with the RNA chaperone Hfq, destabilized *vanT* mRNA and modulated expression of VanT-regulated genes. Unexpectedly, expression of all four *qrr* genes peaked at high cell density, and exogenously added *N*-acylhomoserine lactone molecules induced expression of the *qrr* genes at low cell density. The phosphotransferase VanU, which phosphorylates and activates VanO, repressed expression of the Qrr sRNAs and stabilized *vanT* mRNA. A model is presented proposing that VanU acts as a branch point, aiding cross-regulation between two independent phosphorelay systems that activate or repress expression of the Qrr sRNAs, giving flexibility and precision in modulating VanT expression and inducing a quorum-sensing response to stresses found in a constantly changing aquatic environment.

## Introduction

Quorum sensing is a cell-to-cell communication mechanism that is mediated by small diffusible signal molecules and that is utilized by bacteria to modulate gene expression in response to population density (for review see Ng & Bassler, 2009). Bacteria produce signal molecules, such as *N-*acylhomoserine lactones (AHLs), which accumulate as the cell number increases. When the concentration of the signal molecules reaches a certain threshold, gene expression is activated or repressed by quorum-sensing systems coordinating bacterial activities as a population, which may provide a selective advantage in natural environments.

**Fig. 1.**
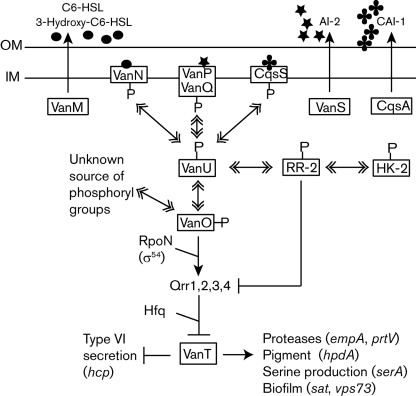
*V. anguillarum* quorum-sensing phosphorelay model. Solid lines with arrows and bars indicate activation and repression of gene expression, respectively. Solid lines with double arrowheads indicate the transfer of phosphoryl groups from one protein to another. Details of the model are given in the Introduction and Discussion. In this study, a model is presented suggesting that the phosphotransferase VanU phosphorylates two response regulators: one response regulator, VanO, activates Qrr sRNA expression, repressing VanT expression, and a second, predicted response regulator (RR-2) is hypothesized to repress Qrr sRNA expression, activating VanT expression. The RR-2 is predicted to be phosphorylated via a histidine kinase (HK-2) of an independent two-component pathway. Abbreviations: OM, outer membrane; IM, inner membrane.

The marine fish pathogen *Vibrio anguillarum*, like many other vibrios, contains components for multiple quorum-sensing phosphorelay systems (Milton, 2006). The components of these phosphorelay systems in vibrios are believed to regulate gene expression in response to the population density similarly to the quorum-sensing phosphorelay networks of *Vibrio harveyi* (Ng & Bassler, 2009). A model of the *V. anguillarum* quorum-sensing phosphorelay signalling systems is given in Fig. 1. In *V. anguillarum*, components of three sensory phosphorelay quorum-sensing systems have been identified: VanM/N, VanS/PQ (Croxatto *et al.*, 2004) and CqsA/S (predicted by Henke & Bassler, 2004; identified in the incomplete genome sequence, E. Hjerde, D. L. Milton, & N. P. Willassen, unpublished data). These three systems channel signal responses via a phosphorylation cascade to a single regulatory network (VanU/O) leading to activation or repression of a main transcriptional regulator (VanT).

At low cell densities, signal molecules are at a low concentration and the hybrid sensor kinases, VanN, VanQ and CqsS, autophosphorylate and thus initiate a phosphorylation cascade that converges onto the phosphotransferase VanU, which phosphorylates the σ^54^-dependent response regulator VanO. Upon phosphorylation, VanO is activated and together with the alternative sigma factor RpoN (σ^54^), induces expression of at least one small regulatory RNA (sRNA) Qrr1 (quorum regulatory RNA). The Qrr sRNA, together with the RNA chaperone Hfq, destabilizes *vanT* mRNA, repressing expression of the quorum-sensing transcriptional regulator VanT (Croxatto *et al.*, 2004; Weber *et al.*, 2008).

At higher cell densities, when a certain threshold of signal molecules is reached, *vanT* expression is induced. Three types of signal molecules are produced by the VanM, VanS and CqsA signal synthases. VanM synthesizes both *N*-hexanoyl-l-homoserine lactone (C6-HSL) and *N*-(3-hydroxyhexanoyl)-l-homoserine lactone, which are sensed by their cognate sensor kinase VanN (Milton *et al.*, 2001). VanS produces the autoinducer (AI)-2 signal molecule, a furanosyl borate diester, which binds to the periplasmic protein VanP. The VanP-AI-2 complex, in turn, binds the sensor kinase VanQ (Croxatto *et al.*, 2004; Denkin & Nelson, 2004). The CqsA synthase is predicted to synthesize CAI-1, (S)-3-hydroxytridecan-4-one (Higgins *et al.*, 2007), which binds its sensor kinase CqsS. Binding of the signal molecules to the sensor kinases VanN, VanQ and CqsS inhibits kinase activity, allowing phosphatase activity to predominate, leading to dephosphorylation and inactivation of VanO. Thus, *qrr1* is not expressed while VanT expression is induced, which leads to gene regulation within the quorum-sensing regulon.

VanT is known to regulate positively the expression of two metalloproteases, EmpA and PrtV, pigment production, exopolysaccharide production, biofilm formation and serine biosynthesis, and to negatively regulate the expression of a main component of the type VI secretion system, the haemolysin co-regulated protein (Hcp) (Croxatto *et al.*, 2002; Weber *et al.*, 2009). In addition, these quorum-sensing systems are an integral part of stress response in *V. anguillarum*. The stress response sigma factor RpoS indirectly induces VanT expression during late exponential growth by repressing expression of the RNA chaperone Hfq and thus stabilizing *vanT* mRNA (Weber *et al.*, 2008). Consequently, VanT and RpoS regulate similar cellular functions to coordinate numerous physiological activities for survival in the aquatic environment.

In this study, the roles of VanU and the Qrr sRNAs in the *V. anguillarum* quorum-sensing phosphorelay were investigated. Three additional *qrr* genes were identified and the expression of all four *qrr* genes was positively regulated by VanO and σ^54^. Together with Hfq, the Qrr sRNAs destabilized *vanT* mRNA repressing expression of VanT. Interestingly, expression of all four *qrr* genes peaked during late exponential growth and exogenously added AHL signal molecules activated expression of the *qrr* genes at low cell density. Moreover, the phosphotransferase VanU repressed expression of the four *qrr* genes in a cell-density-independent manner and activated *vanT* expression post-transcriptionally. A model is presented on how VanU may antagonize the VanO-mediated regulation within the quorum-sensing phosphorelay.

## Methods

### 

#### Strains, plasmids and culture conditions.

Bacterial strains and plasmids are listed in Table 1. *V. anguillarum* strains were routinely grown in trypticase soy broth containing 1 % sodium chloride (TSB-1 %) at 24 °C with aeration, or on trypticase soy agar (TSA) grown at room temperature. *Escherichia coli* was routinely grown at 37 °C with aeration in Luria broth (per l: Bacto tryptone, 10 g; Bacto yeast extract, 5 g; sodium chloride, 5 g). Plasmid transfers from *E. coli* to *V. anguillarum* were performed as described previously (Milton *et al.*, 1996). The vibrio selective medium TCBS agar (Difco) containing 10 µg chloramphenicol ml^−1^ was used after conjugation to select against *E. coli*.

**Table 1.** Bacterial strains and plasmids used in the study

Strain or plasmid	Relevant genotype	Reference or source
**Strains**		
*E. coli*		
BL21(DE3)	Protein expression *E. coli* host	Invitrogen
*V. anguillarum*		
NB10	Wild type *V. anguillarum*, serotype O1, clinical isolate from the Gulf of Bothnia	Norqvist *et al.* (1989)
NB12	Cm^r^, NB10 derivative carrying a mutation in the *empA*	Milton *et al.* (1992)
AC10	NB10 derivative carrying an in-frame deletion in *vanT*	Croxatto *et al.* (2002)
AC11	NB10 derivative carrying an in-frame deletion in *vanO*	Croxatto *et al.* (2004)
BW11	NB10 derivative carrying an in-frame deletion in *hfq*	Weber *et al.* (2008)
DM71	NB10 derivative carrying an in-frame deletion in *vanU*	Croxatto *et al.* (2004)
DM88	NB10 derivative carrying a *vanO* gene that encodes an alanine instead of an aspartate at position 56 (D56A)	This study
DM89	NB10 derivative carrying a *vanO* gene that encodes a glutamate instead of an aspartate at position 56 (D56E)	This study
DM105	NB10 derivative carrying an in-frame deletion of *hcp*	Weber *et al.* (2009)
OTR83	NB10 derivative carrying an in-frame deletion in *rpoN*	O’Toole *et al.* (1997)
SQ1	NB10 derivative carrying deletions of *qrr1* and its promoter	This study
SQ2	NB10 derivative carrying deletions of *qrr2* and its promoter	This study
SQ3	NB10 derivative carrying deletions of *qrr3* and its promoter	This study
SQ4	NB10 derivative carrying deletions of *qrr4* and its promoter	This study
SQ5	NB10 derivative carrying deletions of *qrr1*, *qrr2*, *qrr3* and *qrr4* and their promoters	This study
**Plasmids**		
pDM4	Cm^r^, suicide vector with an R6K origin (requires *pir*) and *sacBR* of *B. subtilis*	Milton *et al.* (1996)
pDM4-Qrr1	Cm^r^, pDM4 carrying a *qrr1* mutant allele that deletes the gene and the promoter (72 bp)	This study
pDM4-Qrr2	Cm^r^, pDM4 carrying a *qrr2* mutant allele that deletes the gene and the promoter (91 bp)	This study
pDM4-Qrr3	Cm^r^, pDM4 carrying a *qrr3* mutant allele that deletes the gene and the promoter (92 bp)	This study
pDM4-Qrr4	Cm^r^, pDM4 carrying a *qrr4* mutant allele that deletes the gene and the promoter (109 bp)	This study
pDM4-VanO-D56A	Cm^r^, pDM4 carrying a *vanO* mutant allele that encodes an alanine instead of an aspartate at position 56	This study
pDM4-VanO-D56E	Cm^r^, pDM4 carrying a *vanO* mutant allele that encodes a glutamate instead of an aspartate at position 56	This study
pDM41	Cm^r^, R6K origin, carrying a promoterless RBSII-*gfp*(ASV)-T_0_ gene	Weber *et al.* (2008)
pDM41-qrr1	Cm^r^, pDM41 carrying a *qrr1* : : *gfp*(ASV) transcriptional gene fusion	Weber *et al.* (2008)
pDM41-qrr2	Cm^r^, pDM41 carrying a *qrr2* : : *gfp*(ASV) transcriptional gene fusion	This study
pDM41-qrr3	Cm^r^, pDM41 carrying a *qrr3* : : *gfp*(ASV) transcriptional gene fusion	This study
pDM41-qrr4	Cm^r^, pDM41 carrying a *qrr4* : : *gfp*(ASV) transcriptional gene fusion	This study
pDM41-vanT·TC	Cm^r^, pDM41 carrying a *vanT* : : *gfp*(ASV) transcriptional gene fusion	Weber *et al.* (2008)
pDM41-vanT3·TL	Cm^r^, pDM41 carrying a *vanT* : : *gfp*(ASV) translational gene fusion	Weber *et al.* (2008)
pETZZ_1a	Km^r^, pET-based protein expression vector containing a T7 promoter, coding sequence for a 6×His-tag fused to a double Z-domain, a TEV protease cleavage site, and a multi-cloning site	Bogomolovas *et al.* (2009)
pETZZ_1a-EmpA	Km^r^, pETZZ_1a derivative encoding an N-terminal 6×His-tag and a double Z-domain fused to EmpA	This study

#### PCR conditions, sequencing and DNA techniques.

PCR was performed as described previously (McGee *et al.*, 1996). When a PCR fragment required minimal errors, the high-fidelity KOD polymerase (Novagen) was used. Unless otherwise stated, conditions for various DNA techniques were according to Sambrook *et al.* (1989). Reaction conditions for DNA-modifying enzymes and DNA restriction enzymes were according to the manufacturer’s instructions. Editing of DNA sequences was done using the Genetics Computer Group Sequence Analysis software (Devereux *et al.*, 1984) of the Genetics Computer Group (University of Wisconsin). Database searches were done using the blast program from the National Center for Biotechnology Information. Genomic DNA sequencing was done by Eurofins MWG as part of a genome sequencing project (E. Hjerde, D. L. Milton and N. P. Willassen, unpublished data). The sequence data have been submitted to the DDBJ/EMBL/GenBank databases under accession nos JN585990 for *qrr2*, JN585991 for *qrr3* and JN585992 for *qrr4*.

#### Mutagenesis methods.

Full gene deletions of *qrr1*, *qrr2*, *qrr3* and *qrr4* were made by allelic exchange using the R6K origin-based suicide vector pDM4 as described previously (Milton *et al.*, 1996). The plasmids pDM4-qrr1, pDM4-qrr2, pDM4-qrr3 and pDM4-qrr4, which were used to make the mutants SQ1, SQ2, SQ3 and SQ4, respectively, carry mutant alleles that delete the entire *qrr* gene as well as its promoter. The mutant SQ5, which carries deletions of all four *qrr* genes, was created by performing four successive allelic exchanges of each gene.

Overlap PCR was used to create *vanO* alleles that encode an amino acid substitution of either an alanine or a glutamate for an aspartate at position 56 in VanO. The alleles were cloned into pDM4 creating pDM4-VanO-D56A and pDM4-VanO-D56E. For easier mutant selection, exchange of the two mutant alleles was done in a Δ*vanO* mutant (AC11) that carries a deletion of *vanO* and strains DM88 and DM89 were created, respectively. All mutations were confirmed by sequencing a DNA fragment of the chromosomal locus carrying the mutation that had been amplified by PCR. Primers used to make the alleles are included in Supplementary Table S1 (available with the online version of this paper).

#### Transcriptional *gfp* reporter fusions and GFP assays.

Transcriptional *gfp* reporter gene fusions were created for *qrr2*, *qrr3* and *qrr4* using the R6K-origin-based suicide plasmid pDM41 as described previously (Weber *et al.*, 2008). The DNA sequence (300 bp) directly upstream of each gene was fused to a *gfp* gene that encodes a short-lived green fluorescent protein GFP-ASV with a half-life of 80 min in *V. anguillarum*. The plasmids pDM41-qrr2, pDM41-qrr3 and pDM41-qrr4 carrying the transcriptional gene fusions were inserted into the promoter region of each gene on the chromosome resulting in a duplication of the promoter region leaving the wild type gene intact. Primers used to create the transcriptional gene fusions are given in Supplementary Table S1.

For GFP assays, strains carrying the *gfp* gene fusions were grown overnight at 24 °C in TSB-1 % with aeration. Overnight cultures were diluted to OD_600_ 0.001 in TSB-1 % and incubation was continued for 24 h. During the 24 h growth, OD_600_ was measured at 2, 4, 6, 8, 10, 12, 14, 18 and 24 h to determine growth. For some GFP assays, the cultures were grown until they reached OD_600_ 0.2 or 1.0. To measure fluorescence, a cell number equivalent to OD_600_ 0.2 (1×10^8^ cells ml^−1^) was removed from each culture. Bacterial cells from each sample were pelleted, washed in 20 µl PBS, pelleted again and resuspended in 20 µl PBS. One microlitre was removed to determine c.f.u. Chloroform (3 µl) was added to the sample, which was then vortexed and incubated for 2 min at room temperature. Cell debris was removed by high speed centrifugation. Fluorescence of a 3 µl sample was measured immediately using a NanoDrop ND-3300. Fluorescence units were divided by the c.f.u. to obtain fluorescence relative to cell number. Wild type without a gene fusion was used to determine the background fluorescence and PBS was used as a blank control. Measurements were done in triplicate and the mean was determined.

#### AHL induction of *qrr* gene expression in single cells.

Overnight cultures of the wild type and the Δ*vanO* and Δ*vanU* mutants containing one of the *qrr* : : *gfp* transcriptional gene fusions were grown overnight in TSB-1 % at 24 °C with aeration. The overnight cultures were diluted in 1 ml TSB-1 % to OD_600_ 0.2 (10^8^ cells ml^−1^). The cells were pelleted in a microfuge and washed once in 1 ml TSB-1 %. Each cell sample was diluted in TSB-1 % to a concentration of 100 bacteria ml^−1^ and the final cell number was confirmed by c.f.u. counts. To allow the level of GFP-ASV in the cells to adjust to the low cell density after dilution, the cells were incubated at room temperature for 80 min. Two bacterial samples from each culture were prepared as above. To one tube, C6-HSL signal molecules were added to a final concentration of 10 nM, while no signal molecules were added to the second tube. Since the C6-HSL stock was prepared in acetonitrile, samples were also prepared similarly using solvent alone as a negative control. Both induced and uninduced samples were incubated for 45 min at room temperature to allow expression of GFP-ASV. The cells were pelleted and all but 10 µl of the supernatant was removed. The pellet was resuspended in the remaining 10 µl and applied to a glass coverslip coated with poly-l-lysine. Prolong anti-fade mounting reagent (Invitrogen) was added to the cells and the coverslip was mounted onto a glass slide. Expression of GFP-ASV in the single cells was determined using a Nikon Eclipse 90i microscope. Bacterial cells from the same field of image were visualized using light microscopy to visualize all bacterial cells and fluorescence microscopy to visualize bacteria expressing GFP-ASV. For bright-field images, a Plan Apo VC 60×/1.40 oil objective with differential interference contrast was used. To detect GFP-ASV fluorescence, UV light and an FITC filter was applied with an exposure time of 200 ms. To determine the background fluorescence, the wild type strain without a *gfp* gene fusion was used. All images were processed with real-time deconvolution using the NIS-Elements software. All images were cropped and processed identically in Adobe Photoshop CS2. This experiment was done using three independent cultures of each strain.

#### Western analyses.

Bacterial cultures were grown in TSB-1 % at 24 °C with aeration for various times. Extracellular proteins and cell lysates were prepared from a 10 ml culture as described previously (Weber *et al.*, 2009). c.f.u. counts were used to equalize samples to 5×10^8^ cells µl^−1^ with SDS-PAGE sample loading buffer. Proteins from 5×10^9^ cells were separated using 12.5 or 15 % SDS-PAGE and Western blot analysis was done using Enhanced Chemiluminescence (Amersham Life Sciences) as described previously (Weber *et al.*, 2009). Production of antisera directed against OmpU, VanT and Hcp was as described previously (Wang *et al.*, 2002; Weber *et al.*, 2008, 2009). EmpA was purified and sent to Agrisera AB, Sweden, for polyclonal rabbit antiserum production.

#### EmpA purification.

Part of the *empA* gene that encodes the mature EmpA protein was amplified by PCR using the primers EmpA-Acc651 and EmpA-*Nco*I and cloned into pETZZ_1a, a pET-based 6×His-tag protein expression vector, creating pETZZ_1a-EmpA. The plasmid pETZZ_1a contains a T7 promoter fused to a sequence encoding a 6×His-tag fused to a double Z-domain, an immunoglobulin G-binding domain of protein A from *Staphylococcus aureus*, and a TEV protease cleavage site (Bogomolovas *et al.*, 2009; Tashiro *et al.*, 1997). The resulting gene fusion encodes a mature EmpA fused to an N-terminal 6×His-ZZ-domain-TEV protein tag. *E. coli* BL21(DE3) carrying pETZZ_1a-EmpA was grown in 400 ml LB containing 50 µg kanamycin ml^−1^ at 37 °C to OD_600_ 0.5. At this time, IPTG (0.5 mM final concentration) was added and the culture was shifted to 16 °C and incubated overnight. Cells were pelleted, frozen and then lysed according to Semsey *et al.* (2002). The soluble fraction was mixed with 2 ml 50 % Ni-NTA bead slurry (Qiagen) and the 6×His-ZZ-domain-EmpA protein was purified according to the manufacturer’s instructions. The eluate was dialysed overnight at 4 °C in buffer containing 50 mM NaH_2_PO_4_ pH 8.0, 200 mM NaCl and 10 % glycerol. To cleave the N-terminal 6×His-ZZ-domain tags from EmpA, TEV protease (Invitrogen, 100 µg ml^−1^) plus 1.0 mM dithiothreitol was incubated with the pure protein for 16 h at 30 °C. TEV protease, which contains a 6×His-tag, and the N-terminal EmpA tags were removed from the protein mix with a Ni-NTA column. Protein concentration was measured using the Bradford reagent (Bio-Rad).

#### Real-time quantitative RT-PCR (qRT-PCR).

Bacterial cultures, equivalent to 1×10^8^ cells ml^−1^, were harvested at OD_600_ 1.0 and total RNA was extracted using the RNeasy Mini kit (Qiagen). The iScript one-step real-time qRT-PCR kit with SYBR Green (Bio-Rad) was used to measure transcript levels of *vanT* in various mutants as described previously (Weber *et al.*, 2008). Calculations for mRNA levels were done according to the standard curve method (Larionov *et al.*, 2005), which normalizes the mRNA amounts to that of the 16S mRNA. Each qRT-PCR was done using three independent cultures, the results were averaged and *P*-values were determined.

#### *vanT* mRNA stability assay.

The *vanT* mRNA stability assays were performed as described previously (Weber *et al.*, 2008). Briefly, bacterial cultures were grown in TSB-1 % to OD_600_ 0.2 and 1.0. To stop RNA transcription, rifampicin (200 µg ml^−1^ final concentration) was added to each culture. Before the addition of rifampicin, a 100 µl sample was removed for the zero time point. For mRNA half-life measurements, samples (100 µl) were removed at 2, 5 and 10 min after addition of rifampicin. Total RNA was isolated and mRNA levels were determined using real-time qRT-PCR. The assay was done in triplicate.

#### Northern analysis.

*V. anguillarum* cultures were grown at 24 °C in TSB-1 % to OD_600_ 1.0. RNA was isolated using the RNAzol RT reagent (Molecular Research Center). RNAzol RT allows the isolation of mRNA (>200 bases) and microRNA (<200 bases) in separate fractions. For the Northern analyses, an mRNA-containing and a microRNA-containing fraction were collected following the manufacturer’s instructions. After precipitation, these pellets were resuspended in DEPC-treated water and RNA concentrations were measured using the RiboGreen RNA Quant-it reagent (Molecular Probes).

To detect *vanT* mRNA, 5 µg of the mRNA-containing (>200 bases) RNA fraction was used and to detect the Qrr sRNAs, 3 µg of the microRNA-containing (<200 bases) RNA fraction was used. RNA samples were separated in a 1.2 % (w/v) formaldehyde agarose gel (20 mM MOPS, 5 mM sodium acetate, 1 mM EDTA, 0.24 M formaldehyde) with a formaldehyde running buffer (20 mM MOPS, 5 mM sodium acetate, 1 mM EDTA, pH 7.0, 2.5 M formaldehyde) using 50 V for 3 h. The RNA was transferred to a ZetaProbe GT membrane (Bio-Rad Laboratories) by overnight capillary transfer with 10× SSC (1500 mM sodium chloride, 150 mM sodium citrate). After transfer, RNA was cross-linked to the membrane using UV light. Pre-hybridization (buffer: 7 % SDS, 250 mM sodium phosphate pH 7.2) and probe hybridization (buffer: 7 % SDS, 250 mM sodium phosphate pH 7.2 plus the DIG-labelled probe) were performed at 50 °C for the *vanT* and *qrr* probes and at 68 °C for 16S rRNA and 5S rRNA probes. The DIG-labelled DNA probes were generated using the PCR DIG probe synthesis kit (Roche) and primers listed in Supplementary Table S1. After hybridization, membranes were washed twice for 30 min with wash buffer I (5 % SDS, 20 mM sodium phosphate pH 7.2) and wash buffer II (5 % SDS, 20 mM sodium phosphate pH 7.2). Probe detection was done using the DIG luminescence detection kit (Roche).

#### Protease activity assay.

Cultures were grown to OD_600_ 0.7 and the extracellular proteins in the supernatant were assayed for protease activity using azocasein as substrate, as described previously (Croxatto *et al.*, 2002).

## Results

### Identification of four quorum regulatory RNAs (Qrr) that are positively regulated by VanO and σ^54^

**Fig. 2.**
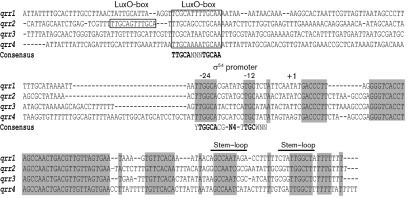
DNA alignment of the four *V. anguillarum qrr* genes and their promoters. The *qrr1*, *qrr2*, *qrr3* and *qrr4* genes and their 5′- and 3′-flanking sequences were aligned using clustal w (Thompson *et al.*, 1994). Regions of the gene sequences with 100 % identity are highlighted in grey. In each promoter region, consensus σ^54^ promoter sequences were found and designated −24 and −12. The consensus σ^54^ promoter sequence (YTGGCACG-N4-TTGCWNN, Barrios *et al.*, 1999) is given below the aligned sites. Putative transcriptional start sites are designated +1. Putative LuxO binding sites were found upstream of the promoters and are indicated with a box. The consensus sequence for the LuxO binding site (TTGCA-N_3_-TGCAA, Lenz *et al.*, 2004) is shown below the aligned sites. Possible stem–loop transcriptional terminators are indicated with solid lines.

The gene *qrr1* in *V. anguillarum*, which encodes the Qrr1 sRNA, was previously found upstream of the *vanOU* operon, which encodes two components of the quorum-sensing phosphorelay system (Weber *et al.*, 2008). Although only one Qrr sRNA is found in *Vibrio fischeri*, multiple *qrr* genes have been found in *Vibrio cholerae* and *V. harveyi* (Lenz *et al.*, 2004; Tu & Bassler, 2007; Miyashiro *et al.*, 2010). Thus, the number of *qrr* genes present in *V. anguillarum* was determined. Using the *qrr1* sequence, a draft of a *V. anguillarum* genome sequence was screened and three additional *qrr* genes, designated *qrr2*, *qrr3* and *qrr4*, were identified. Fig. 2 shows an alignment of the four genes. All *qrr* genes are predicted to be single transcriptional units as stem–loop terminators may be predicted at the 3′-end of each gene. Each promoter region contains a consensus σ^54^ promoter sequence (Barrios *et al.*, 1999) and at least one consensus LuxO box (Lenz *et al.*, 2004), a site to which the LuxO family of σ^54^ activators bind, suggesting that σ^54^ (RpoN) and VanO are required for expression of the Qrr sRNAs.

**Fig. 3.**
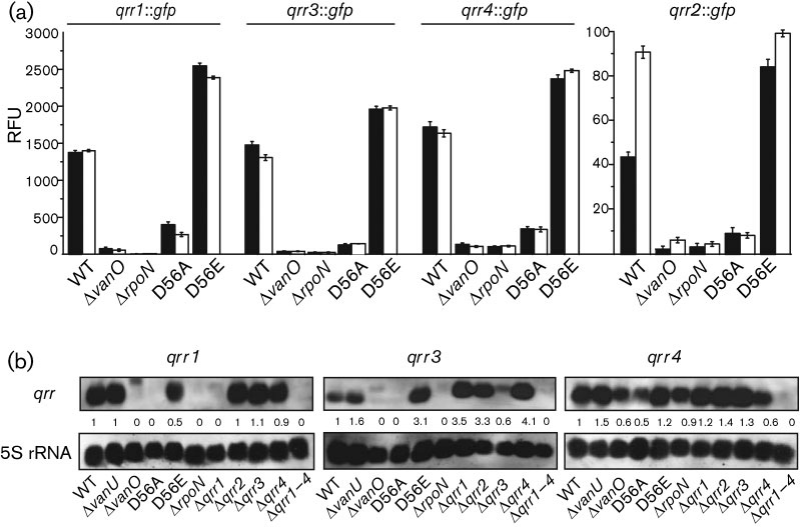
VanO and σ^54^ positively regulate expression of the four Qrr sRNAs. (a) The transcriptional gene fusions *qrr1* : : *gfp*, *qrr2* : : *gfp*, *qrr3* : : *gfp* and *qrr4* : : *gfp* were localized to the chromosome of each strain. Expression of GFP-ASV was measured as fluorescence at early (OD_600_ 0.2, black bars) and late exponential growth (OD_600_ 1.0, white bars) and is presented as relative fluorescence units (RFU; ±sd), which equals fluorescence units per 10^8^ cells. The expression data for each *qrr* : : *gfp* gene fusion are given under a labelled solid line and the *qrr2* : : *gfp* gene fusion data are presented with a different scale from that of the other three gene fusions. (b) Northern analysis of the four Qrr sRNAs. The wild type and various mutant strains were grown to OD_600_ 1.0 and RNA was purified from each strain. For each *qrr* mRNA, 3 µg microRNA-containing (<200 bases) RNA was separated in a denaturing agarose gel. The mRNA was hybridized to a DIG-labelled PCR fragment amplified from a *qrr* gene. The transcript detected on each filter is given above each panel. A probe to detect 5S rRNA transcripts was used as a loading and transfer control. Strains used in both images are designated by their mutation at the bottom of the image.

To test the role of RpoN and VanO in regulating expression of the Qrr sRNAs, transcriptional *qrr* gene fusions were made to a *gfp* gene encoding an unstable GFP, with a half-life of 80 min in *V. anguillarum*. Each *qrr* : : *gfp* transcriptional gene fusion was measured in the wild type and the Δ*vanO* and Δ*rpoN* mutants at OD_600_ 0.2 (low cell density) and OD_600_ 1.0 (high cell density). At both time points, GFP fluorescence for each *qrr* gene fusion was significantly decreased in both mutants compared with the wild type (Fig. 3a). In addition, the *qrr* reporter gene fusions were measured in Δ*vanO* mutants that encode proteins with a single amino acid substitution that is predicted to lock VanO into either an active phosphate-ON state (VanO-D56E) or an inactive phosphate-OFF (VanO-D56A) state (Freeman & Bassler, 1999). Compared with the wild type, GFP expression from all *qrr* : : *gfp* gene fusions was decreased in the inactive VanO-D56A mutant but was increased in the active VanO-D56E mutant. Northern analyses (Fig. 3b) confirm that the Qrr1, Qrr3 and Qrr4 sRNAs are expressed at OD_600_ 1.0 and that VanO and RpoN activate their expression. Although some cross-reactivity occurred with the Qrr4 probe, a decrease in transcripts can be seen for the Δ*vanO* and Δ*rpoN* mutants. Because the *qrr2* transcripts were not detectable using Northern blots, the expression levels of the *qrr2* gene were considered to be much lower than those of the other *qrr* genes. The decreased expression level of *qrr2* may be due to two possible VanO-binding sites detected in the *qrr2* promoter, whereas only one VanO-binding site is predicted in the promoters of the *qrr1*, *qrr3* and *qrr4* genes (Fig. 2).

### Signal molecules induce the expression of Qrr1–4

**Fig. 4.**
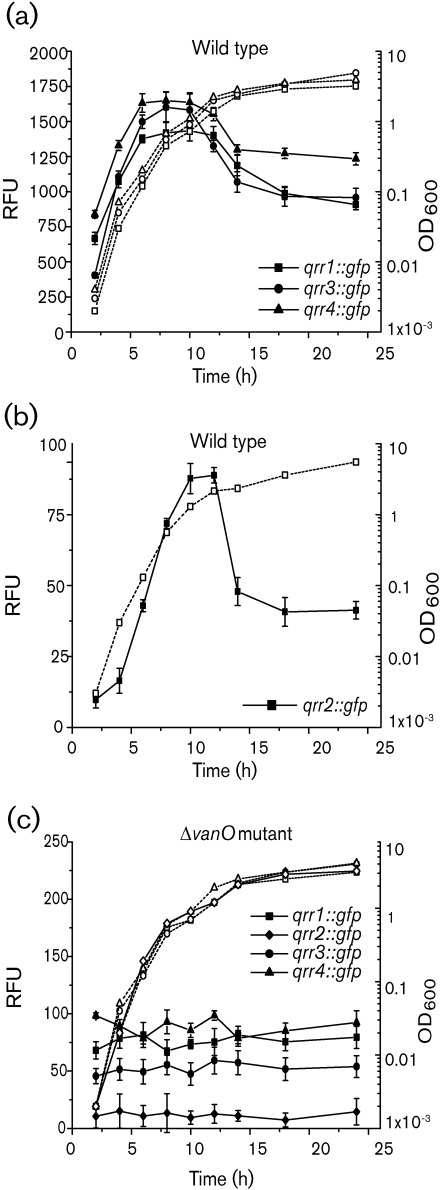
Expression profiles of the *qrr* genes during growth in the wild type and the *vanO* mutant. For the wild type, expression profiles of the transcriptional gene fusions *qrr1* : : *gfp*, *qrr3* : : *gfp* and *qrr4* : : *gfp* are presented in (a), whereas the transcriptional gene fusion *qrr2* : : *gfp* was expressed less than the other *qrr* genes and is presented separately (b). For the *vanO* mutant (c), expression profiles of all *qrr* : : *gfp* transcriptional gene fusions are shown. For all strains, the gene fusions were expressed from the chromosome. Growth (dotted lines) is indicated as OD_600_. At each time point, GFP-ASV expression (solid lines) was measured as fluorescence and presented as relative fluorescence units (RFU; ±sd, which equals fluorescence units per 10^8^ cells.

Interestingly, Fig. 3 indicates that the Qrr sRNAs were expressed at high cell density (OD_600_ 1.0). This observation is in contrast with the expression of the *qrr* genes in *V. harveyi*, *V. cholerae* and *V. fischeri* for which expression peaks during low cell density but decreases significantly at high cell density (Tu & Bassler, 2007; Svenningsen *et al.*, 2008; Miyashiro *et al.*, 2010). This prompted us to determine the expression of the four *qrr* : : *gfp* gene fusions in the wild type and the *vanO* mutant throughout growth. In the wild type, expression profiles of all *qrr* genes were similar, showing a decreased expression at lower cell densities and a peak of expression during entry into the stationary growth phase (Fig. 4a and b). The expression levels of *qrr1*, *qrr3* and *qrr4* were very similar; however, the level of *qrr2* expression was at least 20-fold less throughout growth than the expression of *qrr1*, *qrr3* and *qrr4*. In the *vanO* mutant, a significant loss of expression of all *qrr* genes was observed and no peak of expression was observed as the cell density increased (Fig. 4c) confirming a role for VanO in the cell-density-dependent expression of the *qrr* genes. Moreover, in the wild-type, the expression of all *qrr* genes at the lowest cell density was significantly higher than expression at any time point in the *vanO* mutant, suggesting that expression of the *qrr* genes is induced in the wild type at a lower cell density than was measured for the GFP assays.

**Fig. 5.**
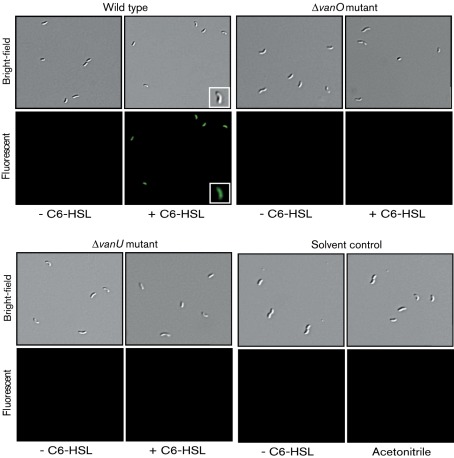
Analyses of the induction of *qrr1* expression by AHL signal molecules in the wild type and the *vanO* and *vanU* mutants. Fluorescence microscopy was used to determine the effect of C6-HSL on expression of the *qrr1* gene in the various strains at a cell density of 100 cells ml^−1^. Overnight cultures carrying the transcriptional gene fusion *qrr1* : : *gfp* on the chromosome were washed and diluted in TSB-1 % to a cell density of 100 bacteria ml^−1^. The cells were allowed to stabilize at low cell density by incubation at room temperature for 80 min, which is the half-life of the unstable GFP-ASV variant in *V. anguillarum*. Two cell samples were prepared for each culture. To one sample (induced), C6-HSL (10 nM) was added, whereas nothing was added to the second sample (uninduced). The bacteria were incubated at room temperature for 45 min and then mounted onto a glass slide. The same field of vision was imaged using both bright-field differential interference contrast microscopy to show all bacteria and UV light to detect GFP-ASV fluorescence, which detected *qrr* gene expression in the bacterial cells. Representative images are given for each strain, which are designated above each group of four images. For the induced sample, one bacterial cell was chosen and enlarged in the inset. +/−C6-HSL at the bottom of a column of images indicates the presence or absence of signal molecules. As the C6-HSL was first dissolved in acetonitrile, solvent alone was used as a negative control. Similar images were also obtained using the gene fusions for *qrr2*, *qrr3* and *qrr4* in the same strains and are presented as Supplementary Figs S1, S2 and S3.

These data suggest that in *V. anguillarum*, the expression of the Qrr RNAs is induced in the presence of the AHL signal molecules instead of repressed, as is shown for other vibrios. If this is true, then GFP fluorescence produced from the *qrr* : : *gfp* gene fusions at a low cell density should increase when AHLs are added to the growth medium exogenously. Since the expression of *qrr1*, *qrr3* and *qrr4* was relatively high at the lowest cell density analysed using a fluorometer, fluorescence microscopy was used to visualize expression of the *qrr* : : *gfp* gene fusions in the wild type and the Δ*vanO* mutant in the presence and absence of AHL signal molecules at a cell density of 100 bacteria ml^−1^. Fig. 5 shows a representative image of single bacterial cells that were analysed for the *qrr1* : : *gfp* gene fusion in the wild type and the Δ*vanO* mutant. At this cell density in the absence of C6-HSL, GFP fluorescence could not be detected in either the wild type or the Δ*vanO* mutant. However, if C6-HSL was added exogenously to the growth medium, an intense fluorescence was observed in the wild type but not in the Δ*vanO* mutant bacterial cells showing that C6-HSL induces expression of the Qrr1 sRNA in the wild type and that this induction is dependent on VanO. Similar results were seen for the *qrr2* : : *gfp*, *qrr3* : : *gfp* and *qrr4* : : *gfp* reporter gene fusions (Supplementary Figs S1, S2 and S3, respectively, all available with the online version of this paper). These data are in sharp contrast with what would be predicted from studies in other *Vibrio* systems and they raise the question as to whether the Qrr sRNAs have a similar function in *V. anguillarum* as they do in other vibrios.

### Qrr sRNAs destabilize *vanT* mRNA repressing expression of VanT

**Fig. 6.**
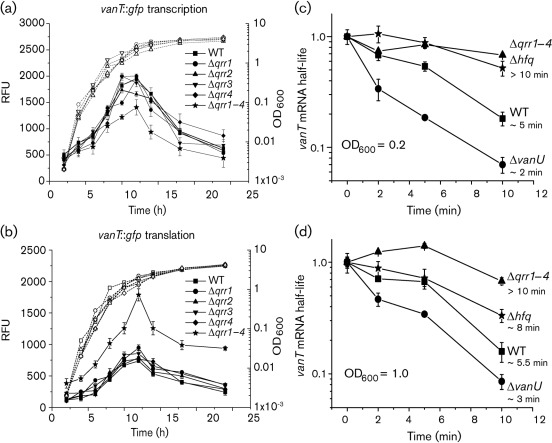
(a, b) Expression of *vanT* during growth of the wild type, and the Δ*qrr1*, Δ*qrr2*, Δ*qrr3*, Δ*qrr4* and Δ*qrr1–4* mutants. The transcriptional (a) and translational (b) *vanT* : : *gfp* reporter gene fusions were expressed from the chromosome and were measured during growth of each strain. Growth (dotted lines) is indicated as OD_600_. At each time point, GFP-ASV expression (solid lines) was measured as fluorescence and presented as relative fluorescence units (RFU), which equals fluorescence units per 10^8^ cells. (c, d) Stability of *vanT* mRNA in the wild-type and the Δ*qrr1–4*, Δ*hfq* and Δ*vanU* mutant strains. All strains were grown to OD_600_ 0.2 (c) and 1.0 (d). At these time points, transcription was stopped by the addition of rifampicin (200 µg ml^−1^). Culture samples were taken at 0, 2, 5 and 10 min. The zero time point was withdrawn before rifampicin addition. Total RNA was isolated from each sample and qRT-PCR was done to determine the amount of *vanT* mRNA remaining at each time point. The mRNA levels at the zero time point for each sample were set at 1.0. The *vanT* mRNA was normalized to the 16S rRNA and each time point was normalized to the respective zero time point. The approximate half-life of the *vanT* mRNA in each strain is given as minutes on the right of each curve.

In *V. cholerae*, *V. harveyi* and *V. fischeri*, in the absence of AHL signal molecules, LuxO homologues are phosphorylated by the quorum-sensing phosphorelay and then activate expression of the Qrr sRNAs, which, together with Hfq, target and destabilize the mRNA of the *luxR* homologues repressing their expression (Lenz *et al.*, 2004; Tu & Bassler, 2007; Svenningsen *et al.*, 2008; Miyashiro *et al.*, 2010). In *V. anguillarum*, Hfq was previously shown to decrease the mRNA half-life of *vanT*, a *luxR* homologue (Weber *et al.*, 2008). Hfq can interact with many different sRNAs to regulate gene expression by either stabilizing or destabilizing mRNA (Gottesman, 2004; Majdalani *et al.*, 2005). To determine if each Qrr sRNA affects the expression of VanT, mutants carrying null deletions of each *qrr* gene (Δ*qrr1*, Δ*qrr2*, Δ*qrr3* and *qrr4*) and one mutant carrying deletions of all four *qrr* genes (Δ*qrr1–4*) were constructed and transcriptional and translational *vanT* : : *gfp* reporter gene fusions were measured in these mutants. Fig. 6(a) and (b) show that deletion of a single *qrr* gene did not affect the expression of *vanT*. Northern analyses (Fig. 3) further show that this lack of an effect on *vanT* expression may be due to an increase in expression of the remaining Qrr sRNAs when one gene is deleted. However, when all four *qrr* genes were deleted, an increase in the translational *vanT* : : *gfp* reporter fusion was observed, which resulted in a decrease in the transcription of *vanT* due to VanT directly repressing transcription from its own promoter (Croxatto *et al.*, 2004). These data suggest that the Qrr sRNAs act redundantly to repress *vanT* expression post-transcriptionally.

To test if the Qrr sRNAs destabilize *vanT* mRNA, the stability of the *vanT* mRNA at OD_600_ 0.2 and 1.0 was measured in the mutant carrying deletions of all *qrr* genes (Δ*qrr1–4*) and in a Δ*hfq* mutant (Fig. 6c and d). At both cell densities, the *vanT* mRNA half-life was approximately 5 min in the wild type, while in the Δ*qrr1–4* and Δ*hfq* mutants, the half-life of *vanT* mRNA increased at least 1.5–2.0-fold showing that the Qrr sRNAs play a role in destabilization of the *vanT* mRNA. Furthermore, these data further confirm that the Qrr sRNAs are expressed and functional at a high cell density.

**Fig. 7.**
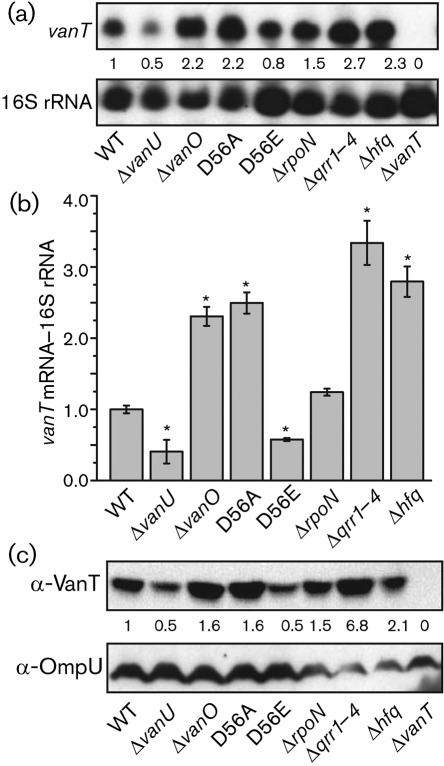
Expression of *vanT* mRNA and VanT protein in the wild type and various mutant strains. (a) Northern analysis of *vanT* mRNA. Total RNA was isolated from cultures grown to OD_600_ 1.0. To detect *vanT* mRNA, 5 µg mRNA-containing (>200 bases) RNA fraction was separated in a denaturing agarose gel and hybridized to a DIG-labelled fragment amplified by PCR from the *vanT* gene. A probe to detect 16S rRNA transcripts was used as a loading/transfer control. The transcript detected on each filter is given to the left of each panel. (b) Real-time qRT-PCR of *vanT* mRNA. Total RNA was isolated from cultures grown to OD_600_ 1.0. Real-time qRT-PCR was done as described in Methods. The *vanT* mRNA was normalized to 16S rRNA transcripts. *P*-values less than 0.001 are considered significant and are indicated by an asterisk. Error bars indicate sd. (c) Western blot analysis of VanT. Culture samples from the wild type and mutant strains were taken at OD_600_ 1.0. Proteins from equal cell numbers were separated by using 12.5 % SDS-PAGE and Western blot analysis was done using a VanT antiserum. An OmpU antiserum was used as a loading/transfer control. After detection of VanT, the blot was stripped and OmpU antiserum was applied. For (a–c), the strains used are indicated by their mutation. For (a) and (c), the *vanT* mutant was included as a negative control.

To show that destabilization of *vanT* mRNA leads to a repressed expression of the VanT transcriptional activator, Northern analyses, qRT-PCR and Western analyses were done using cells grown to a high cell density (OD_600_ 1.0). In addition to the Δ*qrr1–4* and Δ*hfq* mutants, the Δ*vanO* and Δ*rpoN* mutants and the inactive phosphate-OFF (D56A) and the active phosphate-ON (D56E) *vanO* mutants were analysed (Fig. 7). As expected, the inactive *vanO* mutant, the Δ*qrr1–4* mutant and the Δ*hfq* mutant showed a significant increase in *vanT* mRNA and encoded protein, whereas the constitutively active *vanO* mutant (D56E) showed a decrease in *vanT* expression compared with the wild type (Fig. 7). The Δ*rpoN* mutant gave variable results, from no change to an increase in *vanT* expression compared with the wild type, suggesting that RpoN, a global regulator, may affect expression of the *qrr* genes via σ^54^ activators other than VanO. The data are all consistent with the Qrr sRNAs playing a role in the destabilization of the *vanT* mRNA and thus repressing expression of this transcriptional regulator.

### Qrr sRNAs modulate expression of genes regulated by VanT

**Fig. 8.**
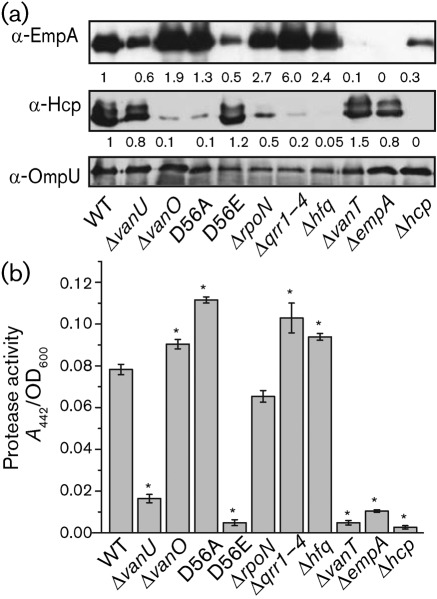
Qrr sRNAs modulate expression of genes regulated by VanT. (a) Western blot analyses of the extracellular proteins EmpA and Hcp. Bacterial cultures were grown for 16 h and supernatant proteins were precipitated with 5 % TCA. Supernatant proteins from equal cell numbers were separated by using 12.5 % SDS-PAGE. Western blot analysis was done using antisera raised against EmpA and Hcp. An OmpU antiserum was used as a loading/transfer control. *V. anguillarum* produces outer membrane vesicles and OmpU is localized within these vesicles and precipitates with the extracellular proteins (data not shown). (b) Total protease activity of extracellular proteins was determined by measuring azocasein degradation. For both (a) and (b), the strains used are indicated by their mutation. For negative controls, the *empA* and the *hcp* mutant were also included. Error bars in (b) indicate sd.

VanT positively regulates expression of the extracellular metalloprotease EmpA (Croxatto *et al.*, 2002) and negatively regulates the expression of Hcp, a protein secreted by the type VI secretion system (T6SS) (Weber *et al.*, 2009). Thus, the Qrr sRNAs may be predicted to modulate expression of EmpA and Hcp indirectly by repressing VanT expression. Western analyses (Fig. 8a) showed that in comparison with the wild type, mutants with a derepressed VanT expression (the inactive VanO-D56A mutant and the Δ*vanO*, Δ*rpoN*, Δ*qrr1-4* and Δ*hfq* deletion mutants) showed an increase in EmpA and a decrease in Hcp protein levels, whereas the active VanO-D56E mutant, which was decreased in VanT expression, and a VanT mutant showed a reverse correlation for both proteins. In addition, total protease activity (Fig. 8b) was measured using spent culture supernatants of the mutants, as VanT activates the expression of a second extracellular metalloprotease PrtV (Weber *et al.*, 2009). When VanT expression was increased or decreased in the mutant strains, the total protease activity also increased or decreased, respectively. Thus, an increase in VanT levels in these mutants strongly correlates with an increase in EmpA, which is positively regulated by VanT, and a decrease in Hcp, which is negatively regulated by VanT. However, the Δ*hcp* mutant did not express EmpA and was decreased in total protease activity. These results were not unexpected since Hcp, which makes up part of the T6SS, was previously shown to modulate the expression of VanT and thus the expression of EmpA (Weber *et al.*, 2009).

### VanU activates *vanT* expression post-transcriptionally by repressing expression of the *qrr1–4* genes

The data presented support the hypothesis that the Qrr sRNAs function to destabilize *vanT* mRNA, as is the case for VanT homologues in other vibrios. However, these data still do not explain how an increase in signal molecules results in an increase in Qrr sRNAs. From studies in *V. harveyi* (Timmen *et al.*, 2006; Neiditch *et al.*, 2005, 2006), the signal molecules are believed to inhibit kinase activity of the hybrid sensor kinases, allowing phosphatase activity to predominate. Thus, VanO is inactivated by dephosphorylation resulting in loss of expression of the *qrr* genes and induction of the quorum sensing regulon. One possible explanation for this observation is that VanU represses expression of the *qrr* genes via an unknown mechanism as well as inducing their expression through the activation of VanO.

**Fig. 9.**
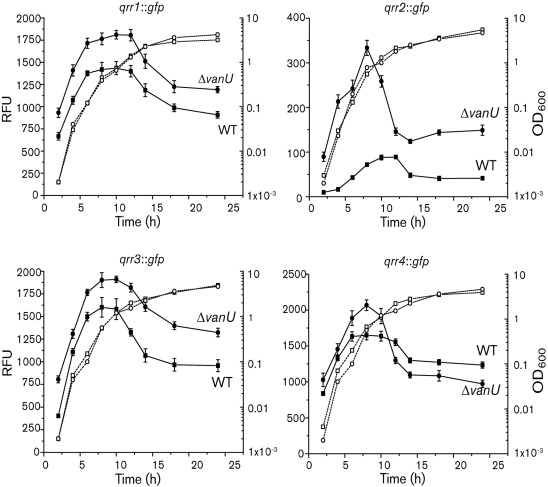
VanU negatively regulates the four *qrr* genes. The transcriptional gene fusions *qrr1* : : *gfp*, *qrr2* : : *gfp*, *qrr3* : : *gfp* and *qrr4* : : *gfp* (indicated above each graph) were expressed from the chromosome and were measured during growth of the wild type and the *vanU* mutant. Growth (dotted lines) is indicated as OD_600_. GFP-ASV expression (solid lines) was measured as fluorescence and is presented as relative fluorescence units (RFU), which equals fluorescence units per 10^8^ cells. Error bars indicate sd.

The four *qrr* : : *gfp* reporter gene fusions were assayed in a Δ*vanU* mutant throughout growth and compared with expression in the wild-type (Fig. 9). Expression of all four *qrr* genes was increased in the Δ*vanU* mutant while maintaining a cell-density-dependent peak of expression. In particular, expression of *qrr2*, the least expressed of the *qrr* genes, was significantly increased in the Δ*vanU* mutant. Northern analyses (Fig. 3) also showed an increased expression of the *qrr3* and *qrr4* mRNA levels in the Δ*vanU* mutant compared with the wild type, confirming that VanU represses expression of these Qrr sRNAs. However, the *qrr1* mRNA appeared to be unaffected and this may reflect the variability of the expression of the *qrr* genes that we have observed in the Δ*vanU* mutant. These data suggest that VanU represses expression of the *qrr* genes using a mechanism independent of the quorum-sensing phosphorelay and that VanU may not be required to activate VanO, which is required for expression of the *qrr* genes. To determine if VanU is needed for a response to signal molecules, the expression of the *qrr* genes was measured in the Δ*vanU* mutant at a cell density of 100 bacteria ml^−1^ in the presence and absence of the C6-HSL signal molecule (Fig. 5, Supplementary Figs S1–S3). Interestingly, in the Δ*vanU* mutant, the *qrr* genes were not expressed in either the presence or absence of signal molecules, similar to results in the Δ*vanO* mutant, suggesting that VanU is needed to activate VanO at this low cell density; however, at higher cell densities, VanO may be phosphorylated by other mechanisms, since the cell-density expression of the *qrr* genes was unaffected in the *vanU* mutant.

An increase in Qrr sRNA production in the Δ*vanU* mutant correlated with a decrease in the half-life of *vanT* mRNA in the Δ*vanU* mutant (2–3 min) compared with the wild-type (5 min) further confirming that the Qrr sRNAs aid destabilization of the *vanT* mRNA (Fig. 6c and d). Consequently, *vanT* mRNA and VanT protein levels were decreased in the Δ*vanU* mutant compared with the wild type (Fig. 7). The decrease in VanT expression in the Δ*vanU* mutant correlated with a decrease in EmpA expression and protease activity (Fig. 8).

**Fig. 10.**
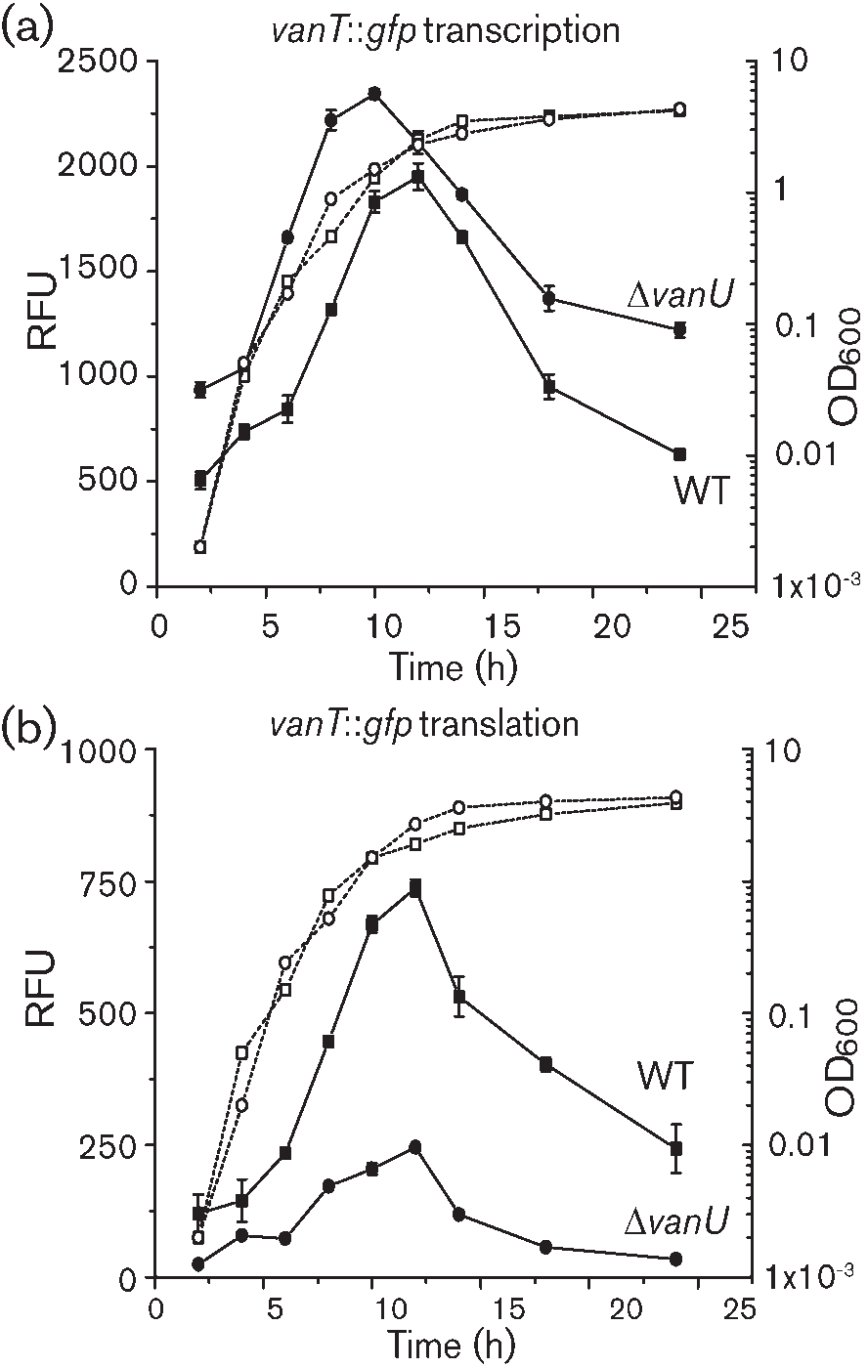
Expression of *vanT* during growth of the wild type and the *vanU* mutant. The transcriptional (a) and translational (b) *vanT* : : *gfp* reporter gene fusions were expressed from the chromosome and were measured during growth of the wild type and the *vanU* mutant. Growth (dotted lines) is indicated as OD_600_. GFP-ASV expression (solid lines) was measured as fluorescence and is presented as relative fluorescence units (RFU), which equals fluorescence units per 10^8^ cells. Error bars indicate sd.

If VanU activates *vanT* expression indirectly through repressing *qrr* gene expression, then the effect of VanU on *vanT* expression may be predicted to occur post-transcriptionally. To test this, *vanT* : : *gfp* transcriptional and translational reporter gene fusions were assayed in the Δ*vanU* mutant throughout growth (Fig. 10). For the translational gene fusion, GFP expression decreased significantly in the Δ*vanU* mutant compared with the wild type. However, an increase in GFP fluorescence was seen for the transcriptional gene fusion in the mutant compared with the wild type, particularly during late exponential growth when *vanT* peaks in expression in the wild type (Weber *et al.*, 2008). As discussed above, the increase in *vanT* transcription is likely due to auto-repression (Croxatto *et al.*, 2004). Taken together, these data show that VanU antagonizes the role of VanO in modulating expression of *vanT* indirectly by repressing expression of the *qrr* genes.

## Discussion

Two-component regulatory systems are used by prokaryotes to sense their environment and to modulate gene expression accordingly (Stock *et al.*, 2000; Mitrophanov & Groisman, 2008). The most common type of two-component signalling systems consist of a histidine sensor kinase, which is an integral inner-membrane protein that senses a specific signal, and a cognate response regulator, whose active state is modulated by the histidine kinase. The histidine kinase responds to a signal and modulates phosphorylation of a response regulator, which in turn modulates gene expression.

Quorum-sensing systems in most vibrios consist of a multi-component phosphorelay signalling system used to sense bacterial populations in an environmental niche (Milton, 2006). These multi-component signalling systems are more complex but are also more versatile versions of the two-component regulatory systems, as they add an additional response regulatory domain and a histidine-containing phosphotransferase domain (Appleby *et al.*, 1996). In vibrios, a common, general theme for the function of these multi-component signalling systems has been established (Ng & Bassler, 2009). The quorum-sensing phosphorelay is initiated via one or more of three hybrid sensor kinases, VanN, VanQ and CqsS (Fig. 1), in the absence of signal molecules at low cell density. The phosphoryl signals transmitted by the sensor kinases converge onto a single phosphotransferease VanU, which phosphorylates and activates the response regulator VanO inducing Qrr sRNA expression. At high cell density, signal molecules accumulate and bind their cognate sensor kinase, inhibiting kinase activity. Phosphatase activity predominates leading to inactivation of VanO and loss of Qrr sRNA expression.

The ancestral *qrr1* gene located upstream of *vanO*, which is found in all sequenced *Vibrio* and *Photobacterium* species is also found in *V. anguillarum* (Weber *et al.*, 2008; Miyashiro *et al.*, 2010). In addition to the ancestral *qrr1* gene, many *Vibrio* species were shown by phylogenetic analysis to contain four or five *qrr* genes (Miyashiro *et al.*, 2010). In this study, four *qrr* genes were identified in *V. anguillarum*. As for other *Vibrio* species, VanO and σ^54^ positively regulated all four *qrr* genes and the Qrr sRNAs together with Hfq destabilized mRNA encoding the global regulator, VanT, which belongs to the HapR family (Lenz *et al.*, 2004; Svenningsen *et al.*, 2009; Tu & Bassler, 2007; Miyashiro *et al.*, 2010). These findings suggest that the multi-component phosphorelay in *V. anguillarum* would activate and deactivate VanO similarly to that in other vibrios. However, Qrr sRNA expression was induced as the cell numbers increased and was repressed at a low cell density, whereas *qrr* expression in other vibrios shows a reverse profile (Tu & Bassler, 2007; Svenningsen *et al.*, 2008; Miyashiro *et al.*, 2010).

In *V. harveyi*, AHL signal molecules bind LuxN and thus inhibit kinase activity of the sensor kinase domain allowing phosphatase activity to predominate (Timmen *et al.*, 2006; Swem *et al.*, 2008; Neiditch *et al.*, 2005, 2006). VanN has high homology to LuxN (Milton *et al.*, 2001). If we assume that VanN functions similarly to LuxN in the presence of AHLs, the signal molecules would be predicted to repress *qrr* expression in *V. anguillarum*. However, AHL signal molecules induced expression of the Qrr sRNAs. Moreover, VanU negatively regulated the *qrr* genes in a cell-density-independent manner antagonizing activation of the *qrr* genes via VanO and stabilizing *vanT* mRNA. If VanN acts as a phosphatase in the presence of signal molecules as LuxN does, then how might VanU repress expression of the Qrr sRNAs while VanO activates expression? One possibility that would explain the data so far is that VanU interacts with a second response regulator that inhibits expression of the Qrr sRNAs and that the second response regulator is part of a distinct, independent two-component system (Fig. 1). Cross regulation such as this between two independent regulatory pathways may provide a physiological benefit to the organism (Laub & Goulian, 2007). In this scenario, VanU becomes a branching point in the phosphorelay signalling system and has the potential to either activate or repress expression of the Qrr sRNAs, possibly in response to various signal molecules, including those for quorum sensing. Such a dual role would also explain the variability we observed in the expression of the *qrr* genes in the Δ*vanU* mutant.

How might VanU work through two response regulators with antagonistic activities to modulate VanT expression? VanU is a detached histidine phosphotransferase (HPt) domain, which may provide an additional point of regulation within the quorum-sensing phosphorelay signalling systems (Croxatto *et al.*, 2004). A detached HPt domain, a common feature amongst eukaryotic but not prokaryotic phosphorelay systems, allows the domain to interact with multiple kinases and response regulators providing greater flexibility in signal transfer (Thomason & Kay, 2000). In addition, evidence from both eukaryotic and prokaryotic systems suggests that HPt domains may have additional functions that provide other means of regulation besides that of transferring a phosphoryl group from one response regulatory domain to another. The HPt domains of the BvgS system in *Bordetella pertussis* and the EvgS system in *E. coli* function to provide specificity between the kinase domains and the cognate response regulatory domains in these signalling pathways (Perraud *et al.*, 1998). Response regulatory proteins are most often activated upon phosphorylation and the cellular response that is controlled by the response regulator is determined by the length of the phosphorylation state (Mitrophanov & Groisman, 2008). Histidine phosphotransferase domains have been shown to aid decay or prolongation of the phosphorylation state, as shown for the ArcB sensor kinase in *E. coli* and YPD1 from *Saccharomyces cerevisiae*, respectively (Georgellis *et al.*, 1998; Janiak-Spens *et al.*, 2000).

YPD1 is the best understood detached HPt domain protein. YPD1 functions to mediate phosphorylation transfer from the histidine sensor kinase SLN1 to two downstream response regulators SSK1 and SKN7 (Li *et al.*, 1998). Thus, YPD1 is a branching point coordinating osmotic stress responses (SSK1) and cell wall biosynthesis and cell cycle control (SKN7). Under normal osmotic conditions, YPD1 has a stronger affinity for interaction with the response regulator domain of SSK1 than that of SKN7. Consequently, YPD1 forms a more stable complex with SSK1 prolonging the phosphorylation state (Janiak-Spens *et al.*, 2000; Porter & West, 2005). When the osmolyte concentrations increase, the YPD1-SSK1~P complex is disrupted leading to dephosphorylation of SSK1 and activation of the osmotic stress response (Kaserer *et al.*, 2009).

As VanU is a detached HPt domain protein, it may interact with VanO and a putative second response regulator to repress *qrr* gene expression. A complex may form between VanU and the second response regulator altering the phosphorylation state of the regulator, which is modulated via an independent sensor kinase that responds to signals that are not cell density related, since VanU represses *qrr* expression in a cell-density-independent manner. One possibility is that VanU may prolong the phosphorylated active state of the second response regulator, leading to a prolongation of the repression of *qrr* gene expression. This model would explain why a *vanU* mutant showed a derepressed *qrr* gene expression and shorter half-life of *vanT* mRNA leading to a decreased VanT expression. This model also suggests that AHL signal molecules, which block the phosphoryl transfers due to kinase activity of VanN, are needed to dephosphorylate VanU and the second response regulator leading to an induced expression of the *qrr* genes. Furthermore, the data suggest that at higher cell numbers, VanO is not dependent on VanU for activation since the *qrr* genes are expressed in a *vanU* mutant. This is not unlikely since in a previous study, VanO was suggested to maintain its activated state in the absence of VanU (Croxatto *et al.*, 2004). The mechanism for how VanU represses *qrr* expression to activate VanT expression is not yet clear. However, the possible involvement of VanU in cross-regulation with an independent pathway that regulates *qrr* expression introduces a new mechanism for modulating, with precision, the expression of VanT, a regulator of stress response in *V. anguillarum*.

In summary, *V. anguillarum* encodes four Qrr sRNAs that are positively regulated by VanO, a response regulator activated by the quorum-sensing phosphorelay system at a low bacterial population. Strikingly different to other vibrios, AHL signal molecules induced expression of the Qrr sRNAs and VanU stabilized *vanT* mRNA by repressing expression of the *qrr* genes. These data suggest that VanU may interact with a second response regulator that is part of an independent regulatory pathway and that represses expression of the *qrr* genes antagonizing the positive regulatory effect of VanO. If this is true, the integration of a putative new branch point downstream of VanU leads to a reversed cellular response of the quorum-sensing phosphorelay system in *V. anguillarum* compared with other vibrio systems. An extra regulatory checkpoint within the phosphorelay may increase the versatility and accuracy of the *V. anguillarum* quorum-sensing signalling system, preventing inappropriate cellular responses and unnecessary use of cellular resources allowing the bacterium to regulate precisely stress responses to a constantly changing aquatic environment. *V. anguillarum*, the oldest known fish pathogen, infects over 50 species of fish worldwide, both wild and cultured, tolerates temperatures in the temperate to subtropic climate zones, and survives in seawater for at least 50 months (Pedersen & Larsen, 1998). The intriguing variation in the *V. anguillarum* quorum-sensing phosphorelay presented in this study may have evolved to give this bacterium the capacity to survive exceedingly well in the highly variable aquatic environment.

## Supplementary Material

Supplementary material
