# Supplementary material for: The phosphotransferase VanU represses expression of four qrr genes antagonizing VanO-mediated quorum-sensing regulation in Vibrio anguillarum
Source: Microbiology (Reading). 2011 Dec;157(Pt 12):3324–39. doi: 10.1099/mic.0.051011-0 (PMC3352281; doi:10.1099/mic.0.051011-0)
Supplement: Supplementary material [file supp_157_12_3324__index.html]

The phosphotransferase VanU represses expression of four qrr genes antagonizing VanO-mediated quorum-sensing regulation in Vibrio anguillarum — Supplementary data 

# The phosphotransferase VanU represses expression of four *qrr* genes antagonizing VanO-mediated quorum-sensing regulation in *Vibrio anguillarum*

## Supplementary data for Weber et al.

**Files in this Data Supplement:**

- Supplementary table
- Supplementary figures
