# Supplementary material for: The phosphotransferase VanU represses expression of four qrr genes antagonizing VanO-mediated quorum-sensing regulation in Vibrio anguillarum
Source: Microbiology (Reading). 2011 Dec;157(Pt 12):3324–39. doi: 10.1099/mic.0.051011-0 (PMC3352281; doi:10.1099/mic.0.051011-0)
Supplement: Supplementary material [file supp_157.12.3324_Supplementary_Figs_051011.pdf]

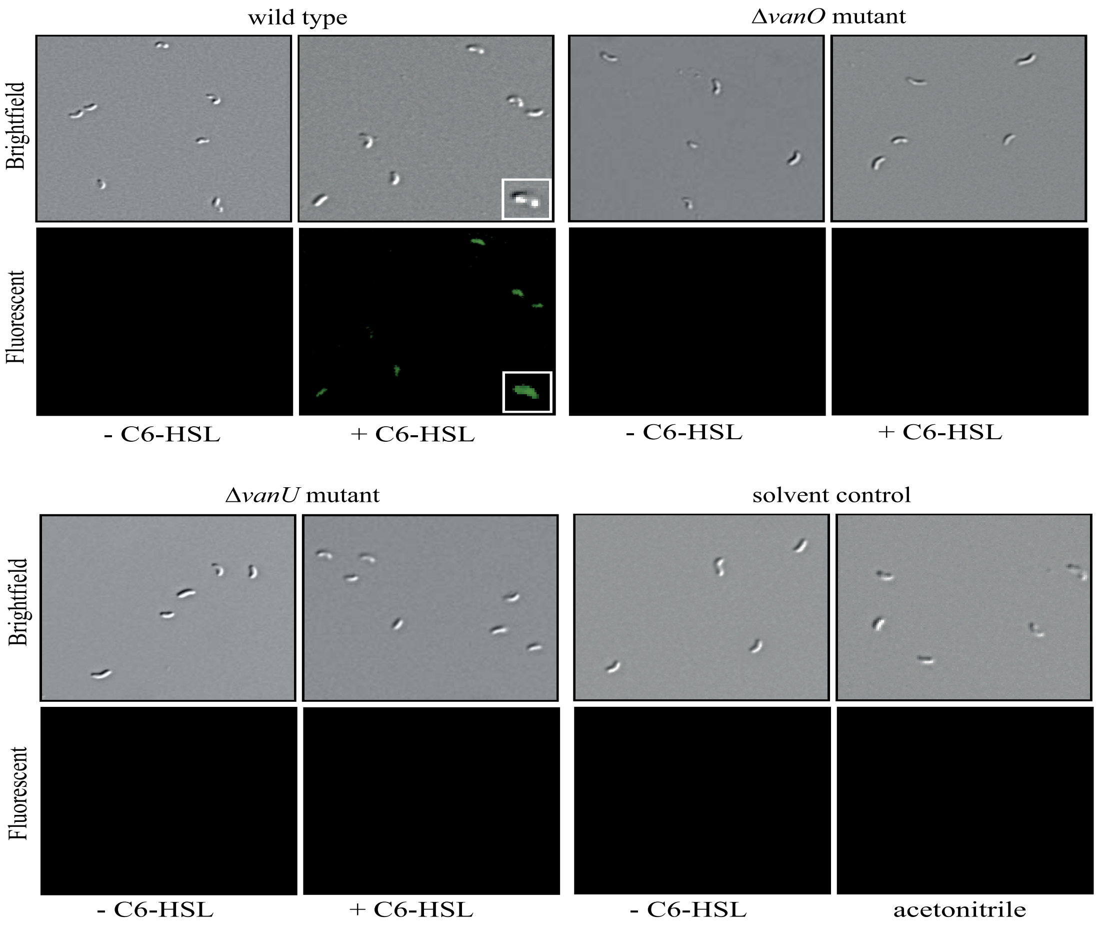

Fig. S1. Analyses of the induction of *qrr2* expression by AHL signal molecules in the wild type and the *vanO* and *vanU* mutants.

Supplementary Fig. S1. Analysis of the induction of *qrr2* expression by AHL signal molecules in the wild type and the *vanO* and *vanU* mutants.

Weber, B., Lindell, K., Elqaidi, S., Hjerde, E., Willassen, N.-P. & Milton, D. L. (2011). The phosphotransferase VanU represses expression of four qrr genes antagonizing VanO-mediated quorum-sensing regulation in *Vibrio anguillarum*. *Microbiology* **157**, 3324–3339.

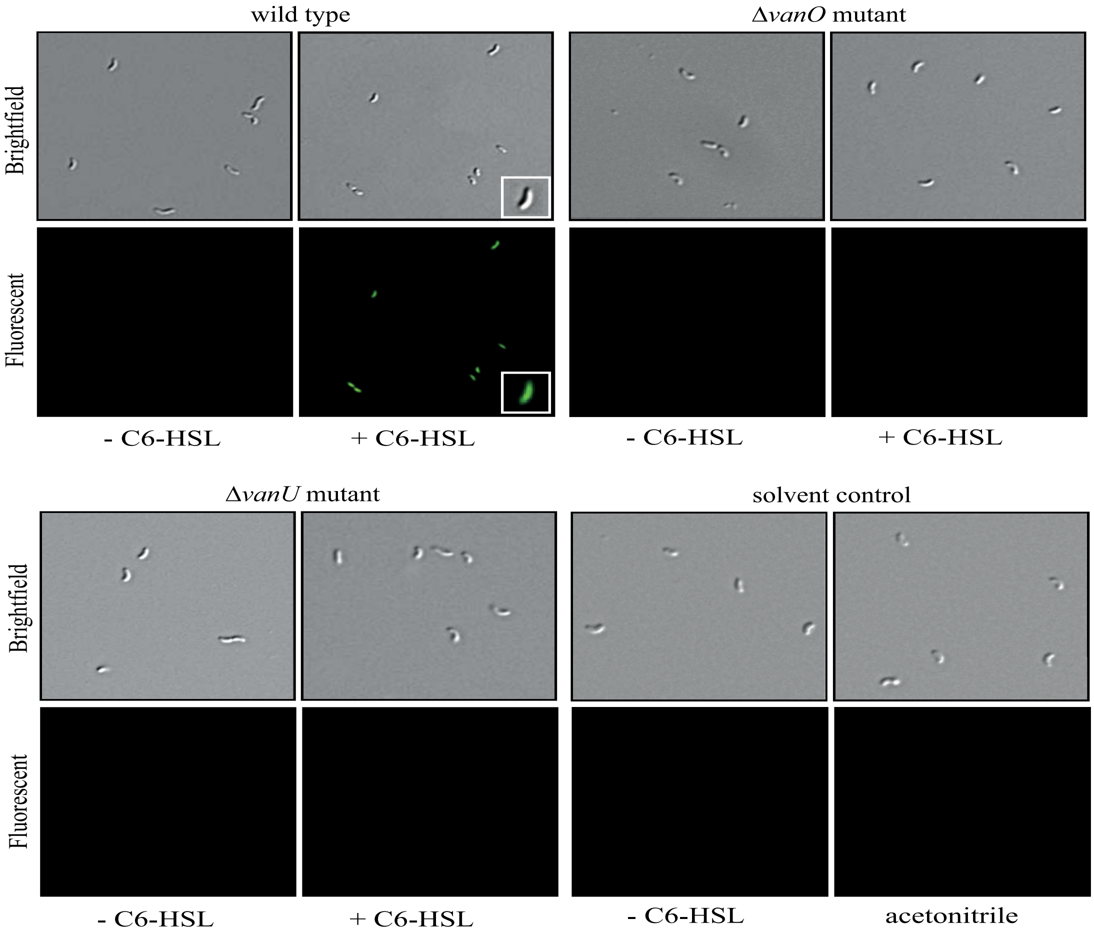

Fig. S2. Analyses of the induction of *qrr3* expression by AHL signal molecules in the wild type and the *vanO* and *vanU* mutants.

Supplementary Fig. S2. Analysis of the induction of *qrr3* expression by AHL signal molecules in the wild type and the *vanO* and *vanU* mutants.

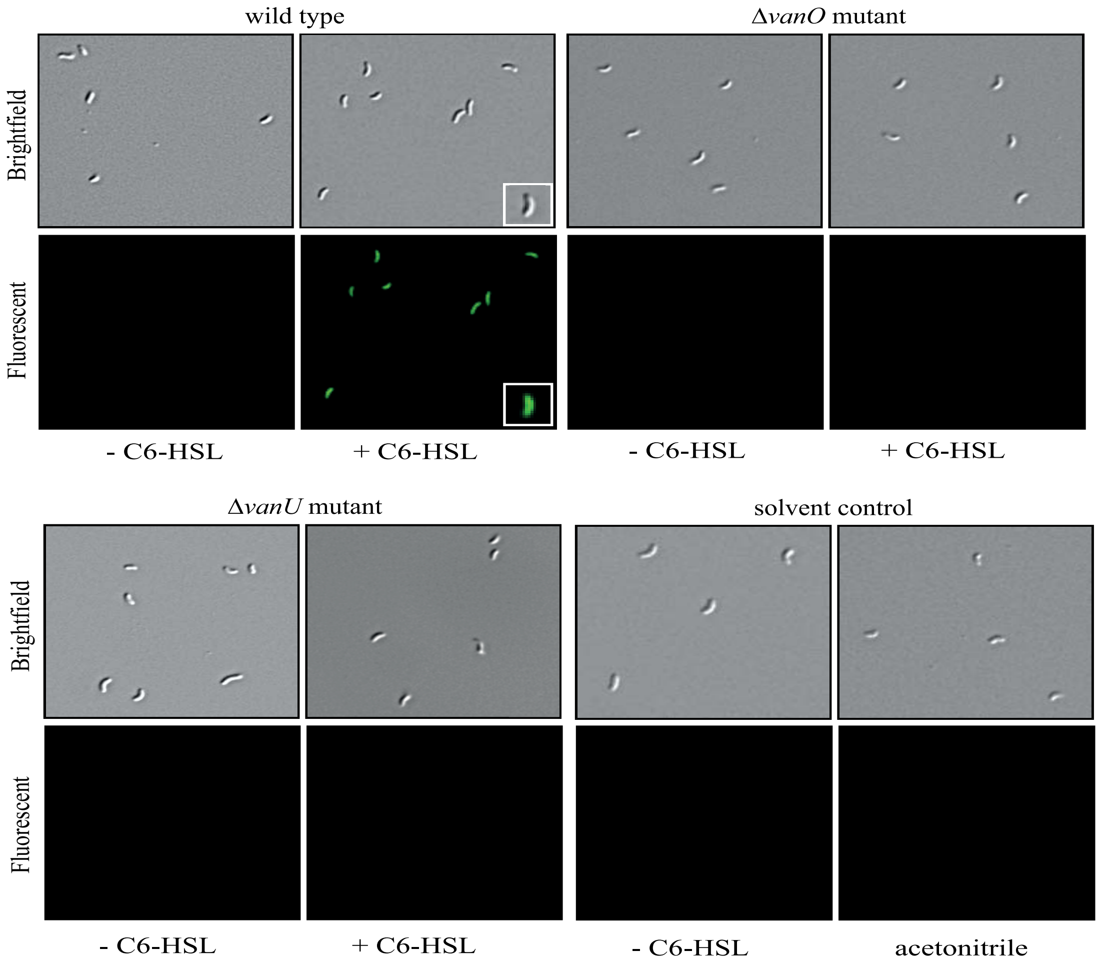

Fig. S3. Analyses of the induction of *qrr4* expression by AHL signal molecules in the wild type and the *vanO* and *vanU* mutants.

Supplementary Fig. S3. Analyses of the induction of *qrr4* expression by AHL signal molecules in the wild-type and the *vanO* and *vanU* mutants.

Weber, B., Lindell, K., Elqaidi, S., Hjerde, E., Willassen, N.-P. & Milton, D. L. (2011). The phosphotransferase VanU represses expression of four qrr genes antagonizing VanO-mediated quorum-sensing regulation in *Vibrio anguillarum*. *Microbiology* **157**, 3324–3339.
