# Supplementary material for: The phosphotransferase VanU represses expression of four qrr genes antagonizing VanO-mediated quorum-sensing regulation in Vibrio anguillarum
Source: Microbiology (Reading). 2011 Dec;157(Pt 12):3324–39. doi: 10.1099/mic.0.051011-0 (PMC3352281; doi:10.1099/mic.0.051011-0)
Supplement: Supplementary material [file supp_157.12.3324_Supplementary_Table_S1_051011.pdf]

Supplementary Table S1. Primers used in this study

Primer name (purpose)	5′–3′ sequence*	Reference gene
**Mutant allele construction**		
Qrr1-A	GGACTAGTTCTAAGGTGGCAGAAAGCTCG	*qrr1*
Qrr1-B	TTGCATAAAATTAAGTGTTCACAAATAAC	*qrr1*
Qrr1-C	TTAATTTTATGCAAAAAGGGCTATTAA	*qrr1*
Qrr1-D	CTCGAGCTCGCCGTATCTTCCACCATC	*qrr1*
Qrr2-A	GGACTAGTTACAGCTAAGTATCGGTT	*qrr2*
Qrr2-B	TAAGCGCTATAAAACATTTACATAGGCCAA	*qrr2*
Qrr2-C	GTTTTATAAGCGCTTAGTTGCTATTGTTTCC	*qrr2*
Qrr2-D	CTCGAGCTCTAATAGATATATGGTGCTGAT	*qrr2*
Qrr3-A	GGACTAGTCGATACGGATCATCCTTATGG	*qrr3*
Qrr3-B	GCAGACCTTTTTTAGCTATATATAAGCCAA	*qrr3*
Qrr3-C	CTAAAAAAGGTCTGCCTTTTTAGCTATGCA	*qrr3*
Qrr3-D	CTCGAGCTCAGTGTATTTGCCGGAAGTATATAA	*qrr3*
Qrr4-A	GGACTAGTAGCCCAGAATTTGATAATGTG	*qrr4*
Qrr4-B	ATTGGCTATTAATAAGTGCTTTAAAACTGCCAA	*qrr4*
Qrr4-C	CACTTATTAATAGCCAATCATACTTTTTGTGAT	*qrr4*
Qrr4-D	CTCGAGCTCGCTCTCTTATTCCAAGACATA	*qrr4*
VanO-H	CTCGAGCTCATCTTGTTATCCACG	*vanO*
VanO-J	GGACTAGTTGCAAAATGCGAACC	*vanO*
VanO-D56-2	AGCAATATGAGGTCCGGCTCTCGG	*vanO*
VanO-D56A-3	GACCTCATATTGCTG***GCT***CTTCGATTACCC	*vanO*
VanO-D56E-3	GACCTCATATTGCTG***GAA***CTTCGATTACCC	*vanO*
***gfp* reporter gene fusions**		
qrr2gfpS	GGTGAGCTCTAACAGTACAAATGG	
qrr2gfpX	TGCTCTAGAATATAGTTATTGCAG	*qrr2*
qrr3gfpS	GGTGAGCTCTCCCTTTTAAAAACG	*qrr2*
qrr3gfpX	TGCTCTAGAAATAGTATTATGCAT	*qrr3*
qrr4gfpS	GGTGAGCTCCCAGTCATTTATGAG	*qrr3*
qrr4gfpX	TGCTCTAGACTTACTATATAGCAG	*qrr4*
**qRT-PCR**		
vanT-left	CCACGCAGATATTGCTGAA	
vanT-right	CTTTCGCATGCAAATCAAGA	*vanT*
16SrRNA-left	CATGCCGCGTGTATGAAGAA	*vanT*
16SrRNA-right	CGTCAAATGATGCTGCTATTAACAA	*16S rRNA*
**Northern analyses**		*16S rRNA*

Weber, B., Lindell, K., Elqaidi, S., Hjerde, E., Willassen, N.-P. & Milton, D. L. (2011). The phosphotransferase VanU represses expression of four qrr genes antagonizing VanO-mediated quorum-sensing regulation in *Vibrio anguillarum*. *Microbiology* **157**, 3324–3339.

VanT-1	CCACGCAGATATTGCTG	*vanT*
VanT-13	ACGTTCAATGGCTTTGAT	*vanT*
27F	AGAGTTTGATCATGGCTCAG	*16S rRNA*
1492R	TACGGYTACCTTGTTACGACTT	*16S rRNA*
Qrr1-RT-FW	CTTAGGGGTCACCTAGC	*qrr1*
Qrr1-RT-REV	AAAGGTCTATTGGCTGTTATTTGT	*qrr1*
Qrr2-1	CTATGTAAATTGTGAACA	*qrr2*
Qrr2-3	CGACCCTTCTTAAGCCGA	*qrr2*
Qrr3-5	TCATTAGACGAGCGAGGA	*qrr3*
Qrr3-6	AATGCGATGCATAGCTAA	*qrr3*
Qrr4-	CTGCTTATGAGAAATGTGC	*qrr4*
Qrr4-4	TGACCCTTCTAAGCCGAG	*qrr4*
**Protein purification**		
EmpA- *Nco*I	GCTACC**ATG**GCAGAGGCGACGGGT	*empA*
EmpA-Acc651	GCTAGGTACCTTAATCCAGTCTTAACGT	*empA*

*Underlined sequences indicate restriction endonuclease sites, bold letters indicate the ATG start codon, and bold, italic letters indicate an amino acid exchange.

Weber, B., Lindell, K., Elqaidi, S., Hjerde, E., Willassen, N.-P. & Milton, D. L. (2011). The phosphotransferase VanU represses expression of four qrr genes antagonizing VanO-mediated quorum-sensing regulation in *Vibrio anguillarum*. *Microbiology* **157**, 3324–3339.
